# Low Seroprevalence of Measles-Specific IgG in Children of Three Ethnic Groups from Mexico: Influence of Age, Sex, Malnutrition and Family Size

**DOI:** 10.3390/vaccines9030295

**Published:** 2021-03-22

**Authors:** Miguel A. Sánchez-Alemán, Ilse A. Gutiérrez-Pérez, Nayeli Díaz-Salgado, Oscar Zaragoza-García, María Olamendi-Portugal, Natividad Castro-Alarcón, Isela Parra-Rojas, Iris P. Guzmán-Guzmán

**Affiliations:** 1 Center of Infectious Diseases Research, National Institute of Public Health, Cuernavaca, Morelos 62100, Mexico; msanchez@insp.mx (M.A.S.-A.); molamendi@insp.mx (M.O.-P.); 2 Faculty of Chemical-Biological Sciences, Autonomous University of Guerrero, Chilpancingo, Guerrero 39087, Mexico; 20389360@uagro.mx (I.A.G.-P.); 15158700@uagro.mx (O.Z.-G.); natividadcastro@uagro.mx (N.C.-A.); iselaparra@uagro.mx (I.P.-R.); 3 Faculty of Natural Sciences, Autonomous University of Morelos, Cuernavaca, Morelos 62209, Mexico; nayely.diaz@uaem.edu.mx

**Keywords:** measles, vaccine, IgG antibodies, children, ethnic groups, Mexico

## Abstract

Background: The reemergence of measles represents a public health problem. The aim of the study was to determine the seroprevalence of IgG antibodies against measles in children of three ethnic groups in southern Mexico and the nutritional status and demographic risk factors associated. Methods: A cross-sectional study in 416 school-age children, 207 belonging to the Tlapaneco ethnic group, 101 to the Mixteco group and 108 were considered Mestizo. Sociodemographic data were collected, an anthropometric evaluation of the children was performed and a fasting blood sample was obtained from each child for the measurement of measles IgG antibodies by Enzyme-Linked Immunosorbent Assay (ELISA). Results: From the total sample, 59% of the children were seropositive for IgG antibodies against measles; in contrast, 41% lacked IgG antibodies. Measles antibody seropositivity was higher in girls (64%). 90.5% of 6-year-old children had higher antibodies seroprevalence, compared to the children between 10 and 13 years old (45.5%). In the three ethnic groups, age was negatively correlated with the index standard ratio (ISR) of measles antibody levels and the families with ≥8 members showed less seropositivity. According to the antibodies levels, most of the positive cases remained around 1 Standard Deviation (SD) of the ISR values and no underweight children had antibody levels above 2 SD. Conclusions: The Anti-Measles serological coverage is low in children of three ethnic groups from Southern Mexico and the age, sex, malnutrition and family size are associated factors. Therefore, it is important to strengthen immunization campaigns, principally in vulnerable groups.

## 1. Introduction

The measles virus belongs to the *Paramixoviridae* family. It is the causal agent of an acute infectious disease, frequently in infancy, characterized by fever, rash, cough, coryza, conjunctivitis, immune suppression and in some cases, central nervous system complications [1]. Measles infections lack specific treatments; however, the prophylactic vaccine is considered the best strategy to prevent this viral infection [2]. Despite the availability of a safe and effective vaccine, in 2018 more than 140,000 people died from measles around the world, mostly children under the age of 5. The trivalent measles-mumps-rubella (MMR) vaccine is recommended to be administered in two doses to ensure immunity and prevent measles outbreaks in children, the first dose applied at 12 months of age and the second when they are six years old [3]. Measles is a world health problem, reemerging in some countries in the Region of the Americas declared free of Measles in 2016 by the Pan-American Health Organization [4]; however, from 1 January 2019 to 24 January 2020, a total of 20,430 confirmed sampling cases and 19 deaths were reported in the region [5]. Moreover, according to the latest update on measles circulated by the European Centre for Diseases Prevention and Control, 29 EU/EEA Member States reported 11, 576 cases of measles, from March 2019 to February 2020, 9168 (79%) of which were laboratory-confirmed. The highest numbers of cases were reported by France, Italy, Bulgaria and Poland [6]. In Mexico, from 2000 to 2019, 185 cases of measles were reported [7]. High vaccination coverage is the most important strategy for preventing the disease. In Mexico, from 1996 to 1999 the coverage reported for the measles vaccine was 97.6% in children from 6 to 10 years old [8] and in 2007, 98.3% of children from 1–4 years old tested positive for measles antibodies, with 99.4% of 5–9-year-old children [9]. However, in 2016 the coverage of the MMR vaccine was only 53.9% in children from 12 to 23 months old, as well as 50.7% in 6-year-old children [10]. This shows a decrease coverage against the measles virus. Mexico has a wide cultural and demographic diversity. Around 62 ethnics groups are present, most of them with low economic, political and social development, which, along with cultural and geographical conditions, increase the health vulnerability of these communities, principally that of children [11]. The reemergence of measles in Mexico is a reality caused by population mobility, low vaccination coverage and other factors. The objective of this study was to determine the serologic coverage for measles as well as to evaluate the nutritional status and associated demographic factors for negative antibodies measles in three groups of children from different ethnic groups in Guerrero, located in southern México.

## 2. Materials and Methods

### 2.1. Study Population

A seroprevalence study was conducted on samples obtained in 2017 from 416 children (214 girls and 202 boys) of 6 to 13 years old, from the state of Guerrero located in southern Mexico, selected by disposition for the participation in this study. 207 children belonged to the Tlapaneco ethnic group, 101 to the Mixteco group and 108 were considered Mestizos. The ethnic groups were defined by the indigenous language spoken by their parents and grandparents, as well as belonging to a community and sharing sociocultural customs. The Mestizo group was made up of children that spoke the Spanish language without an indigenous language antecedent in the family. The parents of children enrolled in basic education centers were invited to participate, together with their child in this study. Mexico reported that 95.7% of the children between 6 and 14 years old, assist school in Guerrero. A questionnaire adapted for this population was applied. All the participants were explained in detail what the study consisted of and were asked for the children’s assent and the signing of the written informed consent from their parents or guardians. The study was approved by the Ethics and Research Committee of Autonomous University of Guerrero (Project identification code: CB-003/2017). The evaluation and sampling were carried out in spaces intended for health-care in the communities by a trained staff.

### 2.2. Data Collection

From a survey carried out on the guardian, sociodemographic data were collected. The interviews were performed by speakers of the original languages, who simultaneously translated the questions and answers during the interview. An anthropometric evaluation was made of the children, determining height with a portable stadiometer (SECA, Hamburg, Germany), weight and body mass index (BMI) using a body composition monitor BF-2000 IRONKIDS (Tanita, Arlington, MA, USA), according to the manufacturer’s recommendations and circumferences of waist and arm using an anthropometric tape with an accuracy of ±0.1 cm (Seca 201, Hamburg, Germany). The weight categories; underweight, normal and overweight, were defined by the criteria of the World Health Organization, considering weight according to age and sex.

### 2.3. Laboratory Measurements

Blood samples were taken by venipuncture after at least 8 h of fasting and were centrifuged to separate the serum and stored at −20 °C for the measurement to measles IgG antibodies. In Mexico, the presence of measles IgG antibodies can be considered related to the vaccine, because the cases of measles are associated with importation. The qualitative determination of measles IgG antibodies in the serum was analyzed using an ELISA kit (HUMAN Worldwide Diagnostics Measles IgG ELISA kit, Wiesbaden, Germany). Test sensitivity/specificity stated by the manufacturer was 91%/100%. The serum was diluted to 1:100 and the absorbance was measured to 690 nm by spectrophotometry in the analyzer Labsystems, Multiskan MS. Positive, equivocal and negative categorization of sera were determined using the cutoff values specified by the manufacturer based on an index standard ratio (ISR). ISR values were defined as follows: ≤0.85, seronegative; 0.85 to 1.14, equivocal; and ≥1.15, seropositive. According to the data, the values of 1 standard deviation (SD), 2SD and 3SD for ISR were calculated among positive values to anti-measles IgG, in order to identify immunization and serologic coverage levels.

### 2.4. Statistical Analysis

A descriptive analysis stratified by ethnic group was performed, defining proportions for categorical variables. The normality of data was tested by Shapiro–Wilk test, so median and 5th to 95th percentiles were used to describe continuous nonparametric variables. The categorical variables were compared by chi-square test. The comparison of quantitative variables among the ethnic groups was performed using the Kruskal–Wallis rank test and relation lineal between quantitative variables was determined by Spearman correlation coefficient (r = rho). The confidence intervals were estimated for each of the variables associated with measles antibodies and a *p* value < 0.05 was considered statistically significant. Statistical analysis was performed with STATA software V.13.0 (StataCorp, College Station, TX, USA) and GraphPad Prism V.8 (GraphPad Software, San Diego, CA, USA).

## 3. Results

### 3.1. Anthropometrical Characteristics According Ethnic Group

The study groups were similar in age and gender. The children were around nine years old, with significant differences in anthropometric measurements between ethnic groups. The height, weight, BMI and arm circumference were significantly larger in the Mestizo group (Table 1); however, among children belonging to ethnic groups the malnutrition by deficit or underweight was more frequent in the Mixteco group. In addition, malnutrition by excess (overweight and obesity) was more frequent in the Mestizo group as shown in Table 1.

### 3.2. Demographic Characteristics According to Ethnic Group

In this study, the employment activity of parents belonging in the Tlapaneco and Mixteco groups was agriculture (around 70%). In addition, around 77% and 73% of children in the Tlapaneco and Mixteco ethnic groups, respectively, refer to household or field work, compared to only 44% of the Mestizo group. According to the family size, in general, families were represented by more than five members per household. In ethnic groups, the principal building material for houses was adobe, while in the Mestizo group it was bricks (Table 2).

### 3.3. Seroprevalence Assessment to Measles Antibodies

**Figure 1:**
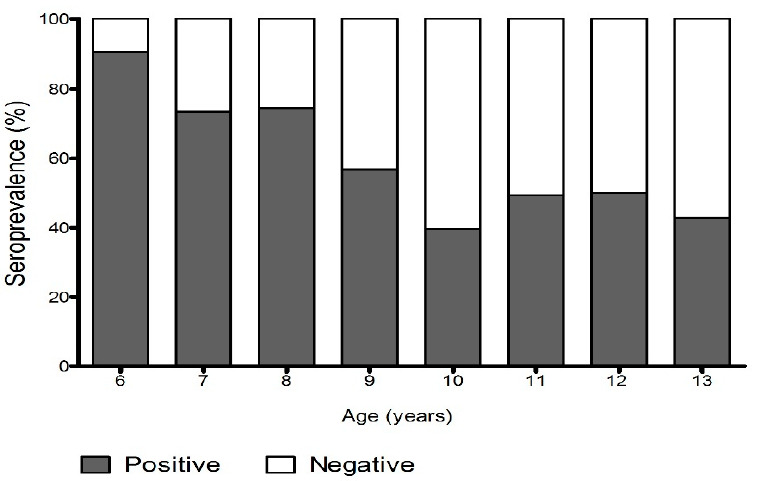
Distribution of positive and negative cases to IgG measles antibodies according to age. Data are reported as proportion (%).

From the total sample of 416 children, 59% (95%CI 54.2–63.6%) showed measles IgG antibodies. In contrast, 41% (95%CI 36.4–45.8%) were IgG antibody negative and therefore could be susceptible to measles infection. The girls had a higher prevalence to the presence of IgG antibodies (64%), as well as children of the Tlapaneco group (63.8%). According to age range, 90.5% of 6-year-old children had measles IgG positive; in contrast, with children from 10 to 13 years old that were only 45.5% positive. In general, families with ≥8 members showed lower seropositivity to measles IgG (52.7%) (Table 3). In Figure 1, it is shown that seropositive cases were more frequent in younger children.

### 3.4. Levels of Measles Antibodies and the Relationship with Age and Weigh Status in the Ethnic Groups

**Figure 2:**
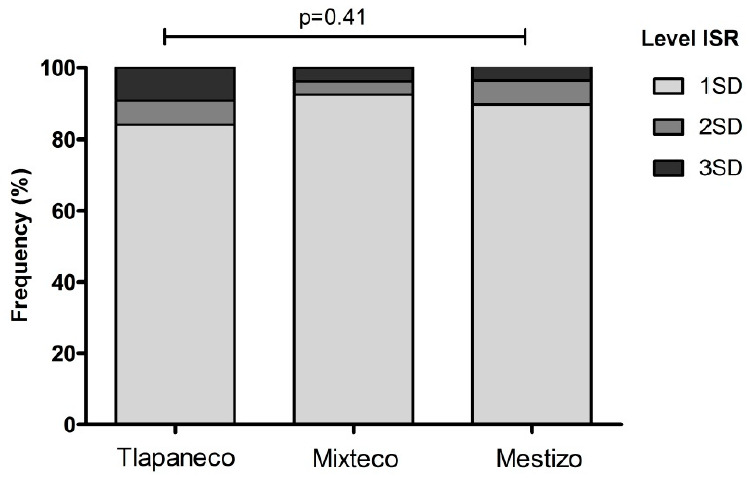
Distribution of positive cases to measles antibodies according to standard deviation (SD) levels of index standard ratio (ISR) in the three ethnic groups. Data are reported as n (%). Chi-square test. Significance value at *p* < 0.05.

**Figure 3:**
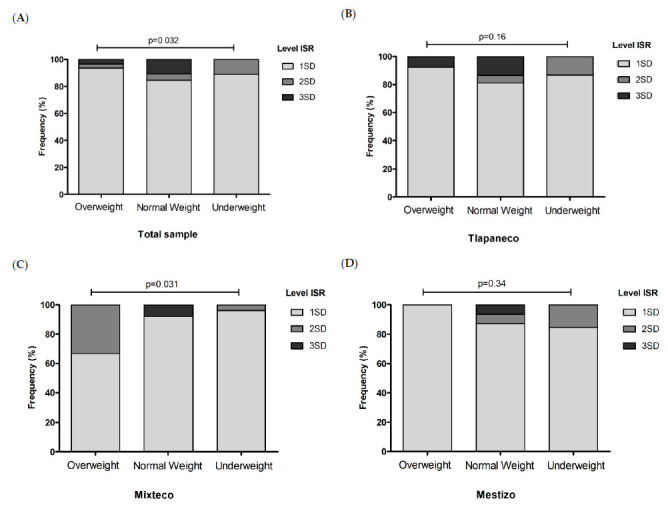
Distribution of positive cases to measles antibodies according to standard deviation (SD) levels of index standard ratio (ISR), considering the weight category. (**A**) Distribution in the total sample. (**B**) Distribution in the Tlapaneco group. (**C**) Distribution in the Mixteco group. (**D**) Distribution in the Mestizo group. Data is reported as *n* (%). Chi-square test. Significance value at *p* < 0.05.

In the three ethnic groups, age was negatively correlated with the ISR levels of measles antibodies (Tlapaneco, r = −0.18, *p* = 0.005; Mestizo, r = −0.28, *p* = 0.003; Mixteco, r = −0.35, *p* < 0.001), as well as the number of members per household in Tlapaneco (r = −0.12, *p* = 0.07) and Mestizo (r = −0.19, *p* = 0.04) groups. As for levels of ISR, the higher value was from Tlapaneco 2.82 (2.5–3.1), followed by Mestizo 2.48 (2.1–2.8) and Mixteco 2.06 (1.7–2.4), *p* = 0.011. However, according to SD levels categories were similar between groups (Figure 2), predominating positive levels between 1SD. According to body weight, no underweight children had antibody levels above of 2SD, in comparison to overweight children and those with normal weight, where the greater serological coverage was found (Figure 3A–D).

## 4. Discussion

Mexico has one of the largest and most diverse indigenous populations, with an estimated 62 communities [11]. However, low socioeconomic development in ethnic groups, as well as cultural and geographic factors, increase the vulnerability of health, principally of children, who have death rates 70% higher in comparison to non-indigenous children [12]. The reemergence of diseases related to viral infections due to the lack of a complete vaccination program represents a serious public health problem in the world.

Between the years 2017–2018, the European Center for Disease Prevention and Control, reported more than 14 thousand cases of measles, with almost 70% of cases located in Italy and Romania [13]. In developed countries, some motives for non-vaccination are parental resistance, religious and moral reasons, skepticism about the effectiveness of vaccines and the use of alternative health care. In other countries, the absence of the disease makes vaccination a victim of its own success, since the benefits of avoiding an almost non-existent disease are not appreciated [14]. This contributes to the risk of measles becoming reestablished, mainly in developing countries where measles coverage vaccination is related to other causes.

In the last decade, the National Health and Nutrition Survey in Mexico reported that the documented coverage for the first dose of MMR vaccination is greater than 80% in children up to 5 years of age. However, coverage with two doses of MMR vaccination (recommended to guarantee coverage of serological immunization) is reported in around 50% of children less than 7 years of age [10,15,16]. In addition, a comparative study reported that the coverage of the complete scheme for MMR decreased 5.3% in the period of 2013–2016 [10]. This shows that around 50% of school-age children do not have immunity against measles. Ruiz-Gómez et al. [9] reported low serological coverage against measles in children with documentation of vaccination, which suggests they are susceptible to acquiring and transmitting the virus contributing to outbreaks of the disease in the community.

In this study, the total serological positivity for antibodies against measles was 59% in children between 6 and 13 years of age; however, the seropositivity varied up to 10.3% among the ethnic groups analyzed, although a statistically significant difference was not found, the lowest frequency of measles antibodies was shown in children of the Mixteco group (53.5%). In 2014, the local Secretary of Health reported that the documented vaccination coverage against MMR in the indigenous population of children from Guerrero was 95.34% [17]. In indigenous populations, the documented vaccination coverage for measles was 83%, while the serologic tests showed 68% [18]. A study in the Mexican children population reported that 30.3% of children received the application of the MMR vaccine after the recommended age, while 14.6% documented no application of the vaccine [19]. However, the low serological coverage in school-age children, as well as discordance between the measurement of coverage by documented records and by serologic markers, demonstrates the potential lack of immunization against measles and the poor efficacy of campaigns of immunization in Mexico. This phenomenon can be partially explained by delayed vaccination, factors related to the cold chain for vaccines or immune-related factors.

In Nahua and Amuzgo children that reside as migrants in Mexican shelters, it was reported that only 25.3% of the children had a complete scheme of vaccination according to age [20]. Among the factors associated with low vaccination coverage in indigenous children are spatial distance to access health services, the male sex of the child and many children in the home or family size. These last two factors were found to be relevant in the present study; therefore, an incomplete vaccination schedule could potentially condition the presence of low titers of anti-measles. In non-indigenous children, the main factors were the lack of or delay in vaccination because of a shortage of health care centers [19].

A national population-based survey reports that maternal illiteracy, maternal age under 20 years, lack of access to health services and speaking an indigenous language are factors associated with low vaccination coverage [10,16]. These places the indigenous population in an environment of susceptibility to the effects of reemerging diseases. Therefore, it is necessary to guarantee effective immunization and individual protection against the measles virus as a prevention strategy for resurgence of endemic transmissions [21,22]. In this study, 6-year-old children had the highest frequency of positive measles antibodies, which could be related to the age at which they received the second dose of MMR vaccine. A meta-analysis study showed that the second dose of MMR vaccine only restores immunity to the level of the first dose [23]. Moreover, antibodies against MMR antigens have been shown to decrease by up to 3% per year [24]. In our population it was observed that antibody levels decrease as age increases, which could also be explained by a cohort effect, in which children aged 10–13 did not receive the vaccine when they were 1 and/or 6 years old. The waning immunity over time could cause the resurgence in measles cases. Against other infections, such as mumps, the application of a third dose of MMR vaccine has been recommended for vulnerable groups [25]. This strategy can be implemented by public health authorities in our population.

In Mexico, it has been described that immunity to measles increases with the number of doses vaccines and that it does not depend on sex, residence or socioeconomic level [9]. In this study, girls presented higher seropositivity and it was observed that age, the presence of malnutrition, as well as the size of the family defined by the number of members per household are factors related to lower seropositivity and levels of measles antibodies against. It is estimated that between 2 and 10% of those vaccinated with two doses of the MMR vaccine fail to develop protective humoral immunity [26]. A longitudinal study reported that measles antibody levels decrease in children who do not receive the second vaccine dose [27]; in addition, the application of the vaccine at an older age could decrease the humoral immune response against vaccination [28]. In concordance with our results, Kizito et al. [29] reported that low serological coverage is associated with nutritional status and poverty, even with the strain used in the measles vaccine. It is recognized that malnutrition related to the lack of minerals such as Zinc, Copper, Selenium and vitamins A and C affects the cellular and humoral components of the immune system [30]. In Mexico and in other countries such as Colombia and Haiti, the vitamin A deficiency is a severe public health problem, principally in indigenous populations and those of Afro-Colombian origin [31]. In Mexico, 19.2% of indigenous children in the south of the country suffer from malnutrition [32] and rural communities, including indigenous populations, report an inadequate supply of nutritional energy content [33], as well as low adherence to the consumption of fruits and vegetables [34]. Therefore, malnutrition could represent a potential factor in the low serological coverage of indigenous populations and their susceptibility to the disease, since malnutrition has been described as increasing the chances of contracting measles by 45% [35]. Malnutrition by deficit or excess related to inadequate intake of nutrients leading to a decreased resistance to infections; in this sense, a low-cost strategy of supplementation with macro and micronutrients would help to support the response to vaccines.

In this study, the IgG anti-measles levels were less in children with underweight. A study shows a relationship between nutritional parameters and the antibodies response to diphtheria, tetanus and measles vaccines in Nigerian children, where well-nourished children had a better response than malnourished children [36]; in our study, normal weight children showed higher levels of IgG anti-measles. An influence of nutrition on the immune response has been shown, as well as the relationship of vaccines-specific response and malnutrition. In malnourished children, components of innate defense against infections (number of dendritic cells and the neutrophils count) tend to be reduced compared with well-nourished children [37] These cells are required to present antigens and trigger the adaptive immune response, so malnutrition could potentially be compromised by the humoral response, not only against measles, but also in other emerging infectious diseases. It has been previously described in pediatric children in Bangladesh that underweight, underdevelopment and emaciation [38] are variables associated to the lack of immunization against measles. Similarly, in children younger than 5 years old that are native to Kenya, it was observed that updated vaccination protects against delayed growth due to the fact that the absence of infections favors the healthy development of children [39]. Poverty and the lack of basic services limit the appropriate nutrition for children, making them vulnerable to the effects related to the deficit of essential health micronutrients.

In Mexico, the risk factors present, together with the political decisions of public health officials in some countries, the migratory effect and other social determinants, a threat to the almost three decades of measles eradication in the country [40]. In 2019, the number of measles cases reported in Mexico increased [41,42]. Currently, Mexico is experiencing a measles outbreak, with 186 cases before 19 June 2020, 77.4% of which were in the capital of the country, 68.8% cases were in unvaccinated people (in addition to 9.7% of children <1 year of age without vaccination) and 21.0% were children 1–4 years old [43]. The population living in Mexico City report good vaccination coverage and potentially fewer barriers than the population of rural areas, highlighting the vulnerability of ethnic groups where living conditions are less favorable. Therefore, it is urgent to implement government strategies to strengthen vaccination and maintain measles eradication in Mexico, mainly in children living in conditions of poverty and vulnerable to poor access to health services, two conditions that would contribute to mortality associated with measles virus infection. In this study, the limitations were that ELISA tests are not the gold standard for the detection of neutralizing antibodies against measles. Specific information on the vaccination record of the population analyzed is lacking, the cross-sectional design does not allow causal relationships and the presence of the volunteer bias is possible.

## 5. Conclusions

In children of three ethnic groups from Southern Mexico the anti-measles serologic coverage is low (59%) and the age, sex, size family and underweight are associated factors. Therefore, it is important to strengthen immunization campaigns considering the current vulnerability of the children.

## Figures and Tables

**Table 1:** Anthropometrical characteristics of the study sample according to ethnic group.

Variable	Tlapaneco (*n* = 207)	Mixteco (*n* = 101)	Mestizo (*n* = 108)	*p* Value
Age, (years) ^a^	10 (6–12)	9 (6–12)	9 (6–12)	0.50
Height, (m) ^a^	1.28 (1.09–1.46)	1.25 (1.08–1.45)	1.31 (1.1–1.52)	0.003 *
Weight, (kg) ^a^	26.7 (17.9–44)	25.2 (17.9–39.2)	28.2 (18.2–53.7)	0.007 *
BMI, (kg/m^2^)	16.5 (14.1–21.2)	15.8 (13.8–20.9)	16.4 (13.9–24.5)	0.015 *
WC, (cm) ^a^	59 (51–73.5)	58 (51–70.5)	59 (51.5–83)	0.144
Arm, (cm) ^a^	18 (14.5–24)	18 (15–21.6)	18.5 (15.3–25.5)	0.021 *
Weight Category ^b^				<0.001 *
Underweight, %	73 (35.3)	46 (45.6)	24 (22.2)	
Normal Weight, %	116 (56.0)	49 (48.5)	56 (51.9)	
Overweight, %	18 (8.7)	6 (5.9)	28 (25.9)	

BMI, Body Mass Index; WC, waist circumference. Data are reported as medians (5–95th percentile) or *n* (%). ^a^ Kruskal-Wallis test. ^b^ Chi-square test. * Significance value at *p* < 0.05.

**Table 2:** Demographic characteristics of the study sample according to ethnic group.

Variable	Tlapaneco (*n* = 207)	Mixteco (*n* = 101)	Mestizo (*n* = 108)	*p* Value
Gender, *n* (%) ^a^				0.97
Male	101 (48.8)	48 (47.5)	53 (49.1)	
Female	106 (51.2)	53 (52.5)	55 (50.9)	
Age category, *n* (%) ^a^				0.35
6	21 (10.1)	7 (6.9)	14 (13)	
7 to 9	80 (38.7)	49 (48.5)	43 (39.8)	
10 to 13	106 (51.2)	45 (44.6)	51 (74.2)	
Parent’s occupation, *n* (%) ^a^				<0.001 *
Farmers	139 (67.2)	70 (69.3)	31 (28.7)	
Traders	14 (6.8)	3 (3)	21 (19.4)	
Others	54 (26.1)	28 (27.7)	56 (51.9)	
House building material, *n* (%) ^a^				<0.001 *
Adobe	76 (36.7)	59 (58.4)	39 (36.1)	
Wood	73 (35.2)	27 (26.7)	20 (18.5)	
Bricks	58 (28.1)	15 (14.9)	49 (45.4)	
Members per household, *n* (%) ^a^				0.04 *
2–4	54 (26.1)	20 (19.8)	27 (25)	
5–7	96 (46.4)	63 (62.4)	63 (58.3)	
≥8	57 (27.5)	18 (17.8)	18 (16.7)	

^a^ Data are reported as *n* (%) by Chi-square test. * Significance value at *p* < 0.05.

**Table 3:** Relationship measles antibodies according to socio-demographic data in total children.

Variable	*n*	Seropositive IgG Anti-Measles% (CI_95_%)	*p* Value
Gender, *n* (%) ^a^			
Male	202	53.5 (46.5–60.4)	0.029 *
Female	214	64.0 (57.5–70.5)	
Age category, *n* (%) ^a^			<0.001 *
6	42	90.5 (81.2–99.7)	
7 to 9	172	66.9 (59.7–73.9)	
10 to 13	202	45.5 (38.6–52.5)	
Ethnic group, *n* (%) ^a^			0.13
Tlapaneco	207	63.8 (57.1–70.3)	
Mixteco	101	53.5 (43.5–63.3)	
Mestizo	108	54.6 (45.1–64.2)	
Weight category, *n* (%) ^a^			0.99
Underweight	143	58.7 (50.5–69.9)	
Normal weight	221	58.8 (52.2–65.3)	
Overweigh	52	59.6 (45.8–73.4)	
Parent’s occupation, *n* (%) ^a^			0.82
Farmers	240	57.9 (51.6–64.2)	
Traders	38	63.2 (47.1–79.2)	
Others	138	59.4 (51.1–67.7)	
House building material, *n* (%) ^a^			0.95
Adobe	174	59.8 (52.4–67.1)	
Wood	120	58.3 (49.3–67.3)	
Bricks	122	58.2 (49.3–67.1)	
Members per household, *n* (%) ^a^			0.36
2–4	101	59.4 (49.7–69.1)	
5–7	222	61.3 (54.8–67.7)	
≥8	93	52.7 (42.3–63.0)	

^a^ Data are presented as *n* and proportions and 95% confidence interval. Chi-square test. * Significance value at *p* < 0.05.

## Data Availability

The data presented in this study are available on request from the corresponding author.
